# Autobiographical Memory, Gratitude, Forgiveness and Sense of Humor: An Intervention in Older Adults

**DOI:** 10.3389/fpsyg.2021.731319

**Published:** 2021-12-14

**Authors:** Alberto Chamorro-Garrido, Encarnación Ramírez-Fernández, Ana Raquel Ortega-Martínez

**Affiliations:** Department of Psychology, University of Jaén, Jaén, Spain

**Keywords:** older adults, forgiveness, gratitude, sense of humor, well-being

## Abstract

Research has shown that happiness and well-being play a fundamental role in the health of older adults. For this reason, programs based on Positive Psychology seek to improve quality of life, preventing and reducing the appearance of emotional disorders. The objective of this study was to verify whether an intervention based on Autobiographical Memory, Forgiveness, Gratitude, and Sense of humor would increase quality of life in institutionalized older adults. We used a quasi-experimental design with pre- and post-intervention measures and follow-on measures at 3, 6, and 12 months. A total of 111 institutionalized older adults participated in the study and were randomly assigned to one of three groups: experimental (*n* = 36), placebo (*n* = 39), and control (*n* = 36). Measurements were taken of depression, subjective happiness, satisfaction with life, psychological well-being, and specific memories. Program duration was 11 weeks, followed by refresher sessions of the activities that had been conducted. The results showed that the intervention was effective, producing lasting increase in the participating adults’ well-being, maintained for the following 12 months, in contrast to the other two groups. In conclusion, the proposed intervention proved to be a novel tool that was effective, easily applied, and able to improve quality of life and emotional disorders in older adults.

## Introduction

Since the late 20th century, aging is no longer being considered an inevitable, universal process of decline, but rather a period where the individual can develop potentialities and resources that were not present in earlier stages of life. The World Health Organization (2002) speaks of the concept of active aging as “a process of optimizing health, participation and safety opportunities with the goal of improving quality of life as people become older,” highlighting that it is not a uniform process and does not take place the same way in every human being.

Successful aging includes three main components: low probability of disease and disease-related disability, high physical and cognitive functional capacity, and active engagement with life. This last component focuses mainly on maintaining interpersonal relations and productive activities. Keeping and even broadening one’s interpersonal relations is a significant determinant of longevity, given that social support – whether emotional or instrumental – can have positive effects on health. The effectiveness of such support will depend on the situation and the individual. On the other hand, productive activity is related to functional capacity, education, and self-efficacy, which together with environmental mastery consistently predict sustained activity in old age (Rowe and Kahn, 1997).

This concept of successful aging, based on objective indicators, has recently been expanded to include positive psychology indicators (Ardelt, 2016). One’s subjective attitude toward the physical and social changes that occur in old age play a crucial role in predicting well-being in this stage of life (Thomsen et al., 2017). Thus, aging is a multidimensional process. There is agreement in the scientific literature that physical, psychological, and social factors must all be addressed in order to age well (Brown et al., 2015).

Positive psychology has been a contributor in this change in the conception of old age. The objective of positive psychology is to increase quality of life and levels of well-being, as well as to mitigate emotional disorders present at different stages of life, using interventions designed to cultivate positive emotions, cognitions, and behaviors (Riva et al., 2012; Baumann et al., 2019).

Ranzijn (2002) stressed the application of positive psychology to the sector of older adults, considering that too much attention had been given to stereotypes about losses and decline in this stage of life, without taking into account possible gains. Positive interventions in older adults can supply useful tools for increasing well-being, happiness, and satisfaction with life, as well as for reducing depressive moods.

Following this idea, different authors have implemented a number of proposed interventions for increasing positive emotions in older adults, training them in one or more human strengths by implementing positive activities that help maintain their skills and resources (see Sutipan et al., 2017, for a review). Based on the results obtained in this line of research, we believe that a particularly interesting intervention would include autobiographical memory, forgiveness, gratitude, and sense of humor. This joint intervention will enhance the effect of the interventions that have been carried out separately. Autobiographical memory plays an important role in the well-being of older adults, because remembering the past induce positive emotions (Latorre et al., 2013). Gratitude helps increase satisfaction with life because it magnifies good memories from the past (Sánchez-Cabaco et al., 2019a). When we forgive, we transform bitterness into neutrality, or even into memories with a positive slant, and so enable greater satisfaction with life (Allemand et al., 2012). Finally, satisfaction increases when good humor increases. A sense of humor is associated with psychological benefits, such as a sensation of happiness, well-being, and harmony (Hirsch et al., 2010).

Training in autobiographical memory is important in old age, because it plays a decisive role in configuring one’s identity and well-being, according to the valence of events recalled. Positive autobiographical memories are associated with greater well-being, in contrast to the high anxiety that is related to negative memories (Merrill et al., 2016). Several studies have revealed that, when presented with a cue that elicits a memory, depressed persons recall less specific, more general memories than do nondepressed individuals, whether the cues are words (Champagne et al., 2016) or images (Ridout et al., 2016).

Life review is an activity based on eliciting specific positive memories; the method is attractive, non-stigmatizing and easily administered (Reker et al., 2014). Interventions based on this therapy suggest that recovering these types of memories can be a protective factor against depression in older adults (Talarowska et al., 2016; Hitchcock et al., 2017), producing increased satisfaction with life (Ricarte et al., 2011) and emotional well-being (Bohlmeijer et al., 2007; Cantarella et al., 2017).

Ramírez et al. (2014) used training in autobiographical memory, gratitude, and forgiveness (MAPEG program) in a sample of institutionalized older adults; they obtained an increase in specific memories, satisfaction with life, subjective happiness, and a reduction in depression and state anxiety. Similarly, Jo and An (2018) used a program based on life review therapy with older institutionalized adults and found a positive impact on satisfaction with life and anxiety toward death. The effectiveness of these interventions corroborates the work of other researchers who used similar approaches (Ortega et al., 2015; Ha et al., 2021).

The practice of gratitude magnifies good memories from the past, helping to increase satisfaction with life. Empirical research shows that gratitude is related to increases in well-being (Barrett-Cheetham et al., 2016; Nezlek et al., 2017); in satisfaction with life, whether considered as a whole (Chen et al., 2016) or in the specific realm of work, health, or relationships with others (Robustelli and Whisman, 2018); in hope and happiness (Witvliet et al., 2019; Bryant et al., 2021), in optimism (Peters et al., 2013); in interpersonal relationships (Sun et al., 2014); and in generosity (Liu and Hao, 2017). Moreover, gratitude is negatively related to depressive symptoms (Senf and Liau, 2013); stress and burnout (Lanham et al., 2012; Cheng et al., 2015); anger (Baxter et al., 2012); anxiety (Petrocchi and Couyoumdjian, 2016); and anhedonia (Simon, 2016).

Méndez et al. (2014) studied gratitude as an existential attitude and its possible relationship to psychological well-being in individuals between the ages of 63 and 96 years. Their results showed that gratitude is significantly related to four of the five dimensions of psychological well-being: self-acceptance, autonomy, purpose in life, and personal growth. Chopik et al. (2019) examined the relationship between gratitude and well-being in different age groups. The results showed that the relationship between gratitude and subjective well-being remained relatively constant throughout life.

Killen and Macaskill (2015) found short-term benefits in both hedonic and eudaimonic well-being after an intervention in gratitude, although after 30 days the benefits only remained in hedonic well-being. Ho et al. (2014) found a decrease in depression scores and an increase in life satisfaction, gratitude, and happiness, through implementing an intervention program that included *optimism*, *gratitude*, savoring, happiness, curiosity, courage, altruism, and meaning of life, and promoted positive experiences in people between the ages of 63 and 105 who lived in senior residences. Ramírez et al. (2014) obtained similar results when using their MAPEG program, which also includes training in gratitude. In this same line, Salces-Cubero et al. (2019) found that gratitude training in persons between the ages of 60 and 89 effected an increase in satisfaction with life, happiness, positive affect, and resilience as well as a decrease in negative affect.

Another interesting line of work for increasing well-being consists of acting on negative memories that generate intense emotions, like resentment and rage, through forgiveness (Robertson and Swickert, 2018). It is a process that has demonstrated value for enrichment and for restoring ties (Silton et al., 2013). Different meta-analyses have shown a robust relationship between forgiveness and psychological and physical health (Lee and Enright, 2019; Rasmussen et al., 2019).

Forgiveness-based interventions enhance social relations (Mooney et al., 2016); relations with one’s partner (Kato, 2016); and kindness (Riaz and Khan, 2016). They are related to an increase in psychological well-being (Cornish and Wade, 2015); in self-esteem (Strelan and Zdaniuk, 2015); in life satisfaction and optimism (Rey and Extremera, 2016); and to a reduction levels of anxiety (Allemand et al., 2013; Silton et al., 2013) and depression (Liao and Wei, 2015; Stackhouse et al., 2016). Other authors have found forgiveness-based interventions to be associated with improvements in anxiety, stress, and physical health (Lin et al., 2013; Stewart et al., 2016; Toussaint et al., 2016). Ermer and Proulx (2016) found that older women who forgave other people were less likely to show symptoms of depression, whether or not they felt forgiven by other individuals, while men showed higher levels of depression when they forgave others but did not feel forgiven.

One source of positive emotions that also regulates negative emotions is sense of humor. Emotional regulation based on a sense of humor is an important personal resource; it is perceived as a coping strategy and a protective factor against adversity (Boerner et al., 2017; Tagalidou et al., 2019).

A sense of humor increases self-esteem (Falkenberg et al., 2011; Goodenough et al., 2012); happiness; and satisfaction with life (Hirsch et al., 2010); it reduces anxiety (Kuiper et al., 2014) and depression (Konradt et al., 2013); emotional and physical wear (Ho, 2017); and it promotes conflict resolution (Campbell and Moroz, 2014) and social relationships, and inhibits stress (Caudill and Woodzicka, 2016; Sánchez et al., 2017).

Tse et al. (2010) investigated the effects of a sense of humor on levels of pain, loneliness, happiness, and life satisfaction in people between the ages of 60 and 92. Their findings showed a decrease in pain and perceived loneliness, and an increase in happiness and satisfaction with life, similar to results obtained by Sánchez et al. (2017). Likewise, Quintero et al. (2015) evaluated the impact of laughter on degree of depression and feelings of loneliness in a group of older adults living in senior residences. Their results showed that the participants’ frame of mind improved significantly, showing greater happiness and less depressed and lonely feelings. Along these lines Santos et al. (2018) carried out a study using laughter therapy with non-institutionalized older adults; their results showed that the intervention led to decreased depression and increased happiness and motivation. Barzegar Bafrooei et al. (2018) found a positive relationship between life expectancy, social support, and sense of humor in older adults.

According to the review carried out by Greengross (2013), several studies in the scientific literature find that as people age, their appreciation of humor increases. This has been considered one of the main defense mechanisms against aging, since it allows emotions to be expressed without unease and without unpleasant effects on others (Harm et al., 2014; Proyer, 2014).

As we have presented in the foregoing, studies on this type of intervention in older adults indicate that benefits are produced (Bolier et al., 2013), even though in most cases they disappear after a time (Smith and Hanni, 2019). This may be because the interventions usually do not last more than 8 weeks, so participants may not be able to incorporate the activities into their daily lives. Consequently, refresher sessions were included in this study as one way to ensure that the individuals will continue to carry out the activities, incorporating them into their daily life, whereby we assume that intervention benefits would be maintained. Moreover, the follow-on period was lengthened in comparison with other studies, to 1 year from the end of the intervention.

Moreover, while many studies assess program effectiveness by identifying the changes found after the intervention, adequate control groups are not always included (Fortuna et al., 2018; Treichler et al., 2020). In the few studies that have used a control group (who received the intervention afterward), placebo groups have not been used. We consider it important to include a group with these characteristics in order to know whether the benefits are due to the positive nature of the training activities.

The objective of the present study, therefore, was to verify (1) whether an intervention in older adults based on training in Autobiographical Memory, Gratitude, Forgiveness, and Sense of humor would be effective in increasing their well-being and (2) whether this increase would be maintained in the long term by introducing follow-on refresher sessions that encouraged further use of these activities in their day-to-day life, and so consolidate a lasting effect. Program effectiveness would also be assessed by comparing the results obtained with a placebo group and a control group.

Specifically, older adults who participated in this intervention were expected to have increased levels of subjective happiness, psychological well-being, life satisfaction, and number of specific positive memories they were able to recall, as well as lower levels of depression and fewer general memories, in comparison with the control and placebo groups, and to their own pre-intervention scores. It was also expected that the effects produced by the intervention would be maintained after 3, 6, and 12 months’ time.

## Materials and Methods

### Participants

The initial sample was composed of 144 institutionalized older adults, of which 66% were women. Participants were randomly assigned to three groups: experimental, placebo, and control. Some participants did not complete every phase of the study, due to physical illness (*n* = 25), change of residence (*n* = 1), death (*n* = 5), and/or voluntary dropout (*n* = 2). None of these persons completed the intervention, only pre-intervention scores were obtained, so their data were not included in the analyses. Pre-intervention scores did not differ between those who continued with the study and those who dropped out. The final sample was thus reduced to 111 participants. Of these, 36 belonged to the experimental group, 39 to the placebo group, and 36 to the control group. The age range of the sample is between 62 and 96 years, the mean age was 83.47 years (SD = 6.78), with the same proportion of men and women as in the initial sample.

The inclusion criteria for study participation were: not showing cognitive impairment [scores of less than 27 on the Mini-Mental Status Examination (MMSE), Spanish version], institutionalized in a senior residence, age 60 or older, and providing informed consent.

### Instruments

*Autobiographical Memory Test* (AMT; Williams and Broadbent, 1986; adapted to Spanish by Serrano et al., 2005). The test uses 5 positive words and 5 negative words, and participants have 60 s to recall a specific memory related to each word. The memories they generate can be specific, categorical, or general. The degree of interjudge agreement (3 judges) for specific positive memories generated in the study sample was 0.79.

*Mini-Mental Status Examination* (Folstein et al., 1975, adapted to Spanish by Lobo et al., 1979). Consisting of 11 items, this instrument assesses eight cognitive areas: place and time orientation, recall (encoding and recent memory), attention-concentration and calculation, comprehensive and expressive language, abstract reasoning, and visuospatial construction. According to the version of the MMSE used in this work, the degrees of cognitive impairment established are as follows: between 30 and 27 points, there is no cognitive impairment; between 26 and 25 points, there could be a possible cognitive impairment; between 24 and 10 points, there is a mild to moderate cognitive impairment; between 9 and 6 points, there is moderate to severe cognitive impairment; and less than 6 points, severe cognitive impairment. We have added this information in the description of the scale. This scale adaptation has a sensitivity of 92% and a specificity of 90%.

*Satisfaction With Life Scale* (SWLS; Diener et al., 1985), adapted by Atienza et al. (2000). The scale evaluates participants’ general levels of well-being with five items on a scale from 1 = “Totally disagree” to 7 = “Totally agree.” Internal consistency obtained in this study was 0.85.

*Subjective Happiness Scale* (SHS; Lyubomirsky and Lepper, 1999 and translated to Spanish by Extremera and Fernández-Berrocal, 2014). It is a 4-item Likert-type scale that measures global subjective happiness using statements whereby the person evaluates himself/herself. The Cronbach alpha coefficient obtained in the sample of these research is 0.89.

*Scale of psychological well-being* (SPWB; Ryff, 1989), adapted to Spanish by Díaz et al. (2006). This instrument measures six dimensions of psychological well-being (Self-acceptance, Purpose in life, Autonomy, Environmental mastery, Positive relationships, and Personal growth). Internal consistency of the subscales in this study sample, measured by Cronbach’s alpha coefficient, was as follows: Self-acceptance = 0.81, Positive relationships with others = 0.79, Autonomy = 0.73, Environmental mastery = 0.70, Purpose in Life = 0.84, and Personal growth = 0.68.

*Geriatric Depression Scale* (Yesavage et al., 1983, adapted to Spanish by Martínez de La Iglesia et al., 2002) is a brief questionnaire in which the older adult is asked to answer 15 yes-or-no items regarding how he/she was feeling during the preceding week. Internal consistency obtained in this study was 0.91.

### Design and Procedure

A mixed factorial design was used. The independent variables were Group, with three levels: experimental, placebo, and control and Time (the different moments when the dependent variables were measured), with five levels: before and after the intervention and at the 3rd, 6th, and 12th month following its completion. The dependent variables were the scores obtained in Depression, Happiness, Satisfaction with life, Psychological well-being, and General and specific memories, both positive and negative.

Once the pertinent authorizations had been obtained from the Ethics Committee for Biomedical Research of Junta de Andalucia (Spain), the study objectives and procedure were explained to the potential participants at each nursing home, also indicating that their data would be recorded and analyzed anonymously and that they could drop out of the research at any time, if they wished to. Finally, they were asked to sign their informed consent.

The people who gave their consent were first assessed with the MMSE (Spanish version) in order to eliminate any participants who showed cognitive impairment. All the participants scored lower than 27, so there was no need to exclude anyone from the study. Next, participants were randomly assigned to one of three groups. In each case, the instruments were administered as an individual interview, with an approximate duration of 60 min.

The experimental group received an intervention focusing on Life Review Therapy, Gratitude, Forgiveness, and Sense of Humor. The intervention consisted of 11 weekly sessions of 60 min each, carried out in a room adapted for this purpose at the residences where the participants lived. Participants were divided into 4 subgroups of 9 participants each, and all were treated by the same professional graduated in Psychology and Master in Positive Psychology. The intervention was administered in person to all participants in a standardized way. Given the characteristics of the sample, this was possible by dividing the intervention group into four small groups.

In the first session to promote group cohesion, we began with an activity in which the participant introduced themselves by saying their name and three positive qualities about themselves. Then, we discussed the importance of being happy during aging.

The second, third, fourth, fifth, and sixth sessions focused on autobiographical memory, life review, and positive emotions in old age. The participants recalled personal stories from their childhood, adolescence, and adulthood, through a process of drawing out their experiences from childhood to the present. Participants were instructed in tasks used to evoke personal experiences through photography (old photos) and touch (recognizing objects from the past through touch) to evoke childhood. To work the adolescence period, we used music (songs that marked their lives) and aromas (capturing memories of their past through different smells). In the case of adult period, the participants were asked to express their memories in an artistic way through drawing, writing, etc.

The seventh session was devoted to gratitude, in this session the benefits of gratitude were explained and an activity was carried out in which each participant told the group things that happened in the last week for which they were grateful and they were also asked to write one thank you letter to a close person.

The eighth session was dedicated to forgiveness. The benefits of forgiveness were explained and an activity was performed consisting of remembering general questions for which they should ask for forgiveness. They were also asked to write a letter or make a phone call to forgive to a person who had been harmed in some way.

The ninth and tenth sessions offered training in sense of humor through mime, telling jokes, making funny gestures, choreography, and comedy videos. What is intended is that through laughter and the positive emotions that are produced by these activities, the participants learn to appreciate humor (laugh at oneself, perceive life from a more positive perspective), use humor in different situations especially in bad moments (positive coping) and also use it to improve their personal relationships.

Finally, the eleventh session was dedicated to summarize what had been done in all the previous sessions and insist in that they continue to carry out the activities learned.

At the end of each session, they were asked to carry out some activity during the week related to their training (homework); the following session began by discussing these activities and resolving any questions that might have come up.

In the case of the placebo group, the first session was the same as that of the experimental group. In the second session, they were given a brief introduction to positive psychology and were asked to spend 10 min before going to sleep, 2 days a week, to reflect on early experiences that may have influenced their adulthood. These experiences were discussed in a group with the psychologist. This work dynamic was maintained during the same period of time that the intervention lasted in the experimental group.

One week after the end of the intervention, all participants were re-evaluated individually through an interview using the same scales. Subsequently, refresher sessions were held 2 weeks after the intervention, and in the 1st, 3rd, 6th, 8th, and 12th month following the intervention, in both the experimental and placebo groups. The objective of these sessions was to promote lasting benefits from the intervention. The need to continue following the guidelines established during the intervention was explained to the older adults in the experimental group: for example, trying to guide their memory toward positive events, being grateful, eliminating feelings of resentment, and encouraging good humor. The refresher sessions were carried out at the same time in the placebo group, with the same dynamics that were used before. Finally, 1 week after the corresponding refresher session, in the 3rd, 6th, and 12th months, measurements were taken to assess whether the benefits obtained were lost over time.

In the case of the control group, all assessments were carried out at the same times as in the other groups, but without intervening in any way. Participants in this group, like those in the placebo group, were placed on a waiting list to receive the intervention if the program was shown to be effective.

### Statistical Analyses

Descriptive statistics were calculated for the different dependent variables, as well as Pearson product-moment correlation coefficients between them using Hmisc library (Harrell, 2021). In addition, the data were analyzed using the general linear model (library stats; R Core Team, 2021), taking the Group variable as the inter-subject factor and the Time variable as the repeated measures factor with the five levels described. *A posteriori* comparison was made using the Tukey HSD test (implemented in the postHoc library, Labouriau, 2020). All statistical tests were conducted using R proyect for statistical computing and statistical decisions were made at a significance level of 0.05.

## Results

Descriptive statistics for the different dependent variables in the three groups are shown in Tables 1–3. Table 4 describes the correlations found between the different measurements.

**Table 1:** Descriptive statistics for the dependent variables depression, happiness, and satisfaction with life in the three groups over time.

	Pre	Post	3 months	6 months	12 months
Depression					
Experimental					
*M*	3.93	1.93	2.69	2.24	3.41
SD	0.65	0.56	0.62	0.60	0.66
Placebo					
*M*	3.41	4.25	5.00	4.56	5.92
SD	0.56	0.48	0.54	0.52	0.57
Control					
*M*	4.72	4.55	5.03	5.86	5.69
SD	0.58	0.50	0.56	0.54	0.60
Happiness					
Experimental					
*M*	4.46	5.28	5.29	5.31	4.64
SD	0.24	0.22	0.25	0.22	0.28
Placebo					
*M*	4.56	4.53	4.50	4.36	3.94
SD	0.21	0.19	0.21	0.18	0.21
Control					
*M*	4.32	4.14	4.00	4.03	3.85
SD	0.22	0.19	0.22	0.19	0.22
Satisfaction with life					
Experimental					
*M*	23.80	27.14	26.31	26.69	25.34
SD	0.99	0.88	0.96	0.92	0.96
Placebo					
*M*	24.82	22.02	23.20	22.15	20.10
SD	0.86	0.76	0.83	0.79	0.83
Control					
*M*	23.94	23.39	22.64	22.36	21.50
SD	0.89	0.79	0.86	0.83	0.86

**Table 2:** Descriptive statistics for the different dimensions of psychological well-being in the three groups over time.

	Pre	Post	3 months	6 months	12 months
Self-acceptance					
Experimental					
*M*	25.24	31.41	27.69	27.69	27.31
SD	0.90	0.89	1.00	1.01	1.04
Placebo					
*M*	26.72	27.87	27.72	26.64	24.66
SD	0.77	0.77	0.87	0.88	0.90
Control					
*M*	26.33	26.39	26.56	25.92	25.89
SD	0.80	0.80	0.90	0.91	0.94
Environmental mastery					
Experimental					
*M*	26.27	30.83	28.83	29.52	27.93
SD	0.93	0.79	1.01	1.01	1.09
Placebo					
*M*	27.56	26.36	25.49	26.00	24.02
SD	0.80	0.68	0.87	0.87	0.94
Control					
*M*	27.03	26.05	25.31	26.47	25.33
SD	0.84	0.70	0.91	0.91	0.98
Personal growth					
Experimental					
*M*	24.21	27.10	27.86	26.45	25.93
SD	1.25	1.20	1.45	1.27	1.34
Placebo					
*M*	24.05	22.26	23.67	22.28	20.15
SD	1.08	1.03	1.25	1.10	1.16
Control					
*M*	22.97	22.44	21.86	20.0.6	21.55
SD	1.12	1.07	1.30	1.14	1.21
Purpose in life					
Experimental					
*M*	20.10	26.38	23.90	23.93	22.55
SD	1.15	1.08	1.25	1.20	1.24
Placebo					
*M*	22.33	20.97	19.87	20.36	18.31
SD	0.99	0.93	1.08	1.04	1.07
Control					
*M*	20.25	20.53	18.80	19.78	18.06
SD	1.03	0.97	1.12	1.08	1.11
Positive relationships with others					
Experimental					
*M*	22.96	24.93	25.17	24.90	25.24
SD	1.31	1.33	1.35	1.23	1.35
Placebo					
*M*	26.05	23.72	23.95	23.10	22.95
SD	1.13	1.15	1.16	1.06	1.17
Control					
*M*	22.50	22.11	21.22	21.33	20.58
SD	1.18	1.19	1.21	1.10	1.21
Autonomy					
Experimental					
*M*	30.93	31.76	34.00	32.45	32.45
SD	1.31	1.41	1.28	1.45	1.53
Placebo					
*M*	33.15	33.56	34.87	31.92	32.92
SD	1.13	1.25	1.10	1.25	1.32
Control					
*M*	33.94	35.72	34.58	33.19	34.86
SD	1.17	1.26	1.15	1.30	1.37

**Table 3:** Descriptive statistics for the different memories in the three groups over time.

	Pre	Post	12 months
General positive memories			
Experimental			
*M*	1.47	2.44	1.00
SD	0.17	0.24	0.18
Placebo			
*M*	1.74	2.20	1.87
SD	0.16	0.23	0.17
Control			
*M*	1.11	2.55	1.58
SD	0.17	0.24	0.18
Specific positive memories			
Experimental			
*M*	1.83	2.68	2.89
SD	0.22	0.22	0.23
Placebo			
*M*	1.84	1.87	1.46
SD	0.21	0.21	0.22
Control			
*M*	2.03	1.80	1.28
SD	0.22	0.22	0.23
General negative memories			
Experimental			
*M*	1.22	1.11	1.08
SD	0.17	0.17	0.18
Placebo			
*M*	1.03	0.87	1.41
SD	0.16	0.16	0.17
Control			
*M*	0.97	1.53	1.42
SD	0.17	0.17	0.18
Specific negative memories			
Experimental			
*M*	1.28	1.68	1.39
DT	0.16	0.16	0.16
Placebo			
*M*	1.28	1.31	1.26
DT	0.15	0.16	0.15
Control			
*M*	1.17	1.17	0.97
DT	0.16	0.16	0.16

**Table 4:** Pearson’s correlation coefficients found between depression, happiness, satisfaction with life, and the six dimensions of psychological well-being at all time points.

	1	2	3	4	5	6	7	8
*Pre-intervention scores*								
Depression (1)	–							
Happiness (2)	−0.752^**^	–						
Satisfaction with life (3)	−0.749^**^	0.713^**^	–					
Self-acceptance (4)	−0.625^**^	0.616^**^	0.668^**^	–				
Environmental mastery (5)	−0.653^**^	0.578^**^	0.525^**^	0.641^**^	–			
Personal growth (6)	−0.532^**^	0.484^**^	0.462^**^	0.442^**^	0.480^**^	–		
Purpose in life (7)	−0.608^**^	0.593^**^	0.541^**^	0.539^**^	0.596^**^	0.480^**^	–	
Positive relationships with others (8)	−0.385^**^	0.310^**^	0.405^**^	0.428^**^	0.426^**^	0.481^**^	0.437^**^	
Autonomy (9)	−0.309^**^	0.207^*^	0.411^**^	0.366^**^	0.253^**^	0.258^**^	0.342^**^	0.221^*^
*Post-intervention scores*								
Depression (1)	–							
Happiness (2)	−0.707^**^							
Satisfaction with life (3)	−0.660^**^	0.661^**^						
Self-acceptance (4)	−0.560^**^	0.579^**^	0.495^**^					
Environmental mastery (5)	−0.591^**^	0.578^**^	0.545^**^	0.496^**^				
Personal growth (6)	−0.399^**^	0.493^**^	0.309^**^	0.226^*^	0.421^**^			
Purpose in life (7)	−0.579^**^	0.575^**^	0.529^**^	0.473^**^	0.526^**^	0.444^**^		
Positive relationships with others (8)	−0.394^**^	0.493^**^	0.455^**^	0.402^**^	0.372^**^	0.209^*^	0.420^**^	
Autonomy (9)	−300^**^	0.276^**^	0.221^*^	0.052	0.206^*^	0.246^**^	0.258^**^	0.303^**^
*3 months*								
Depression (1)	–							
Happiness (2)	−0.834^**^							
Satisfaction with life (3)	−0.725^**^	0.832^**^						
Self-acceptance (4)	−0.569^**^	0.554^**^	0.617^**^					
Environmental mastery (5)	−0.707^**^	0.700^**^	0.681^**^	0.560^**^				
Personal growth (6)	−0.640^**^	0.656^**^	0.566^**^	0.453^**^	0.644^**^			
Purpose in life (7)	−0.638	0.704^**^	0.709^**^	0.595^**^	0.674^**^	0.605^**^		
Positive relationships with others (8)	−0.437	0.391^**^	0.478^**^	0.447^**^	0.424^**^	0.453^**^	0.464^**^	
Autonomy (9)	−0.268	0.271^**^	0.255^**^	0.232^*^	0.223^*^	0.312^**^	0.339^**^	0.174
*6 months*								
Depression (1)	–							
Happiness (2)	−0.783^**^	–						
Satisfaction with life (3)	−0.797^**^	0.786^**^	–					
Self-acceptance (4)	−0.580^**^	0.566^**^	0.703^**^	–				
Environmental mastery (5)	−0.721^**^	0.674^**^	0.759^**^	0.585^**^	–			
Personal growth (6)	−0.611^**^	0.620^**^	0.562^**^	0.401^**^	0.607^**^	–		
Purpose in life (7)	−0.675^**^	0.680^**^	0.665^**^	0.543^**^	0.713^**^	0.656^**^	–	
Positive relationships with others (8)	−0.498^**^	−469^**^	0.446^**^	0.393^**^	0.539^**^	0.504^**^	0.524^**^	–
Autonomy (9)	−0.318^**^	0.310^**^	0.491^**^	0.439^**^	0.366^**^	0.309^**^	0.357^**^	0.300^**^
*12 months*								
Depression (1)	–							
Happiness (2)	−0.799^**^	–						
Satisfaction with life (3)	−0.778^**^	0.812^**^	–					
Self-acceptance (4)	−0.698^**^	0.684^**^	0.697^**^	–				
Environmental mastery (5)	−0.759^**^	0.717^**^	0.678^**^	0.710^**^	–			
Personal growth (6)	−0.555^**^	0.560^**^	0.587^**^	0.455^**^	0.646^**^	–		
Purpose in life (7)	−690^**^	0.686^**^	0.697^**^	0.563^**^	0.671^**^	0.656^**^	–	
Positive relationships with others (8)	−0.469^**^	0.432^**^	0.480^**^	0.425^**^	0.479^**^	0.534^**^	0.513^**^	–
Autonomy (9)	−0.229^*^	0.197^*^	0.232^*^	0.193^*^	0.255^**^	0.284^**^	0.208^*^	0.141

* *p < 0.05*;

** *p < 0.01*.

An exploratory analysis to verify compliance with parametric assumptions revealed that the variables had normal distribution (Shapiro–Wilk test *p* > 0.05) and the Levene test confirmed homoscedasticity in all of them (*p* > 0.05). Consequently, parametric tests were carried out in all cases. Furthermore, for an analysis of differences in the pretest, an ANOVA was computed with each of the measures considered in this study as dependent variables, and the three groups (intervention, placebo, and control) as grouping variable. There was a nonsignificant main effect (all *p* > 0.05) and no significant differences were found between the three groups neither in age or sex.

**Figure 1:**
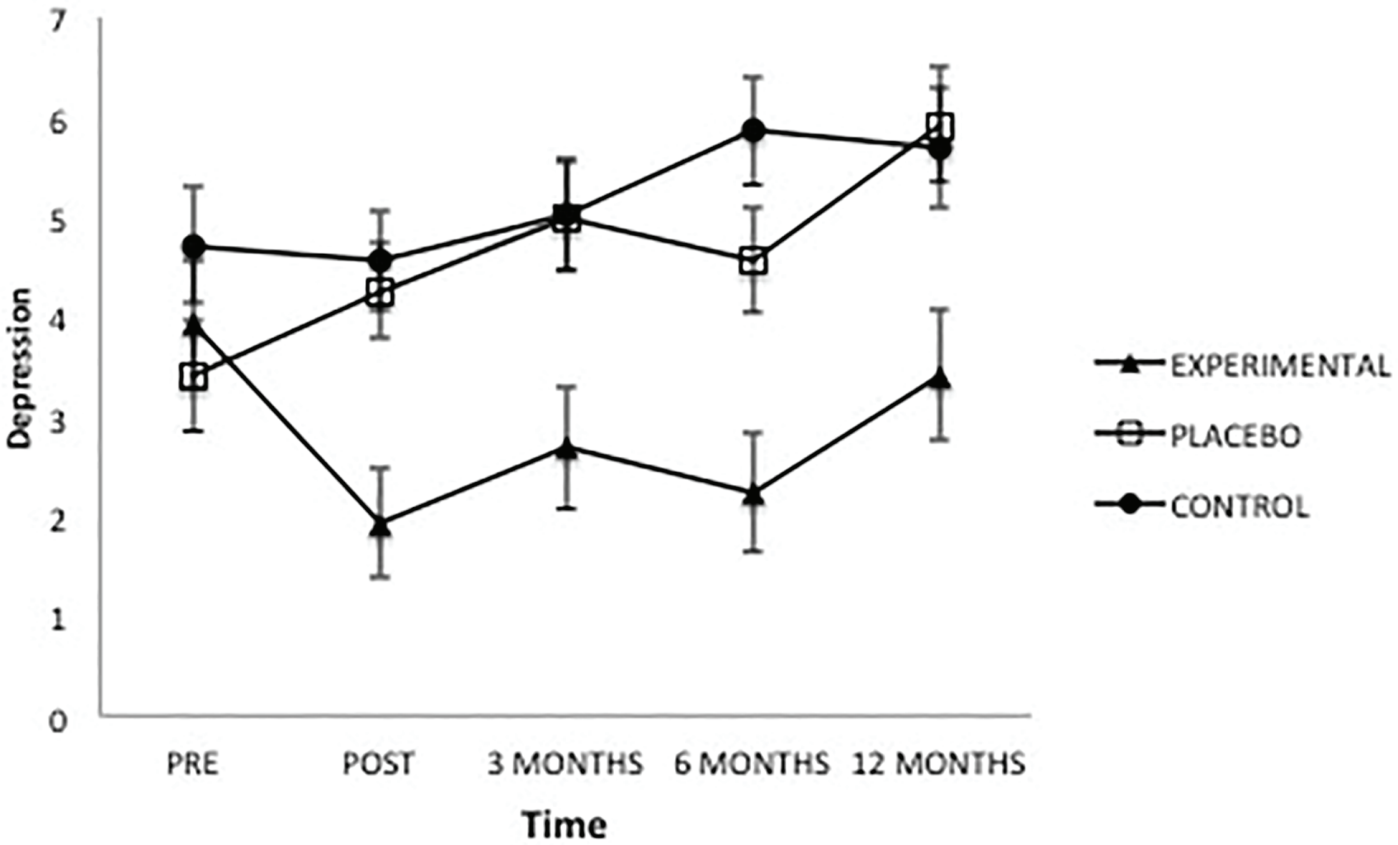
Average scores of experimental, placebo, and control groups in depression as a function of time. The error bars represent the standard error of mean (SEM).

Analysis of the dependent variable “Depression” showed a significant effect from the factor Group *F*(2, 101) = 5.83, *p* = 0.004, $\eta_{p}^{2}=0.10$, from the factor Time *F*(4, 101) = 6.94, *p* = 0.000, $\eta_{p}^{2}=0.10$ and from the interaction between the two *F*(8, 101) = 4.5, *p* = 0.000, $\eta_{p}^{2}=0.10$. Once the intervention was completed, the Experimental group presented significantly lower levels of depression than the Placebo group (*p* = 0.008) and the Control group (*p* = 0.003); between the latter groups there were no differences (*p* = 1.00). As seen in Figure 1, this same result was maintained at 3 months (experimental vs. placebo, *p* = 0.013; experimental vs. control, *p* = 0.014; placebo vs. control, *p* = 1.00), 6 months (experimental vs. placebo, *p* = 0.028; experimental vs. control, *p* = 0.000; placebo vs. control, *p* = 0.258), and 12 months after the intervention (experimental vs. placebo, *p* = 0.012; experimental vs. control, *p* = 0.030; placebo vs. control, *p* = 1.00).

In the Experimental group, moreover, scores obtained before the intervention were significantly different from those obtained afterward (*p* = 0.000) and at 3-month (*p* = 0.010) and 6-month (*p* = 0.001) follow-ups, but there were no differences from scores at the 12-month follow-up (*p* = 0.371). Twelve-month follow-on scores were significantly higher than those obtained at the end of the intervention (*p* = 0.009) and at 6 months (*p *= 0.029). Regarding the Placebo group, the pre-intervention scores were significantly lower than those obtained at the end of the intervention (*p* = 0.020), and at 3 months (*p* = 0.004), 6 months (*p* = 0.034), and 12 months after (*p* = 0.000). Depression showed an increase over time, and scores obtained after 1 year were significantly higher than the rest (post-intervention, *p* = 0.003; 3 months, *p* = 0.049; 6 months *p* = 0.002). Finally, in the Control group, pre-intervention scores showed no differences from the rest of the levels; but significant differences did appear between the different post-intervention measures. Specifically, Depression continued to rise, being higher at 6 months than at 3 months (*p* = 0.04) and at post-intervention (*p* = 0.02); likewise, depression at 1 year was higher than at post-intervention (*p* = 0.04).

**Figure 2:**
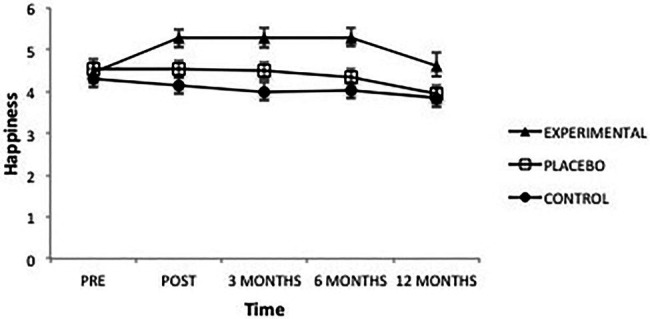
Average scores of experimental, placebo, and control groups in happiness as a function of time. The error bars represent the standard error of mean (SEM).

The scores obtained in “Happiness” showed a significant effect from the factor Group *F*(2, 101) = 6.38, *p* = 0.002, $\eta_{p}^{2}=0.11$, from the factor Time *F*(4, 101) = 6.91, *p* = 0.000, $\eta_{p}^{2}=0.06$ and from the interaction, *F*(8, 101) = 3.66, *p* = 0.000, $\eta_{p}^{2}=0.07$ (Figure 2). Analysis of this interaction showed that, once the intervention was completed, the Experimental group presented significantly higher levels of Happiness than the Placebo (*p* = 0.045) and Control groups (*p* = 0.001); between the latter groups there were no differences (*p* = 0.449). This same result was maintained at 3 months (experimental vs. placebo, *p* = 0.041; experimental vs. control, *p* = 0.001; placebo vs. control, *p* = 0.031), and at 6 months (experimental vs. placebo, *p* = 0.010; experimental vs. control, *p* = 0.000; placebo vs. control, *p* = 0.688); after a year the differences had disappeared.

Similarly, pre-intervention scores in the Experimental group were significantly lower than those obtained afterward (*p* = 0.001) and at 3-month (*p* = 0.000) and 6-month follow-ups (*p* = 0.000). However, 1 year later, Happiness scores returned to initial levels, dropping significantly (*p* = 0.365). In the Placebo group, scores progressively declined, although differences were not significant; only the values obtained after 12 months were significantly different, contrasting with all other levels (pre-intervention, *p* = 0.019; post-intervention, *p* = 0.007; 3 months, *p* = 0.006; and 6 months, *p* = 0.008). Finally, in the Control group, differences were found only between the pre-intervention scores and the scores after 1 year, where the latter scores were lower (*p* = 0.007).

**Figure 3:**
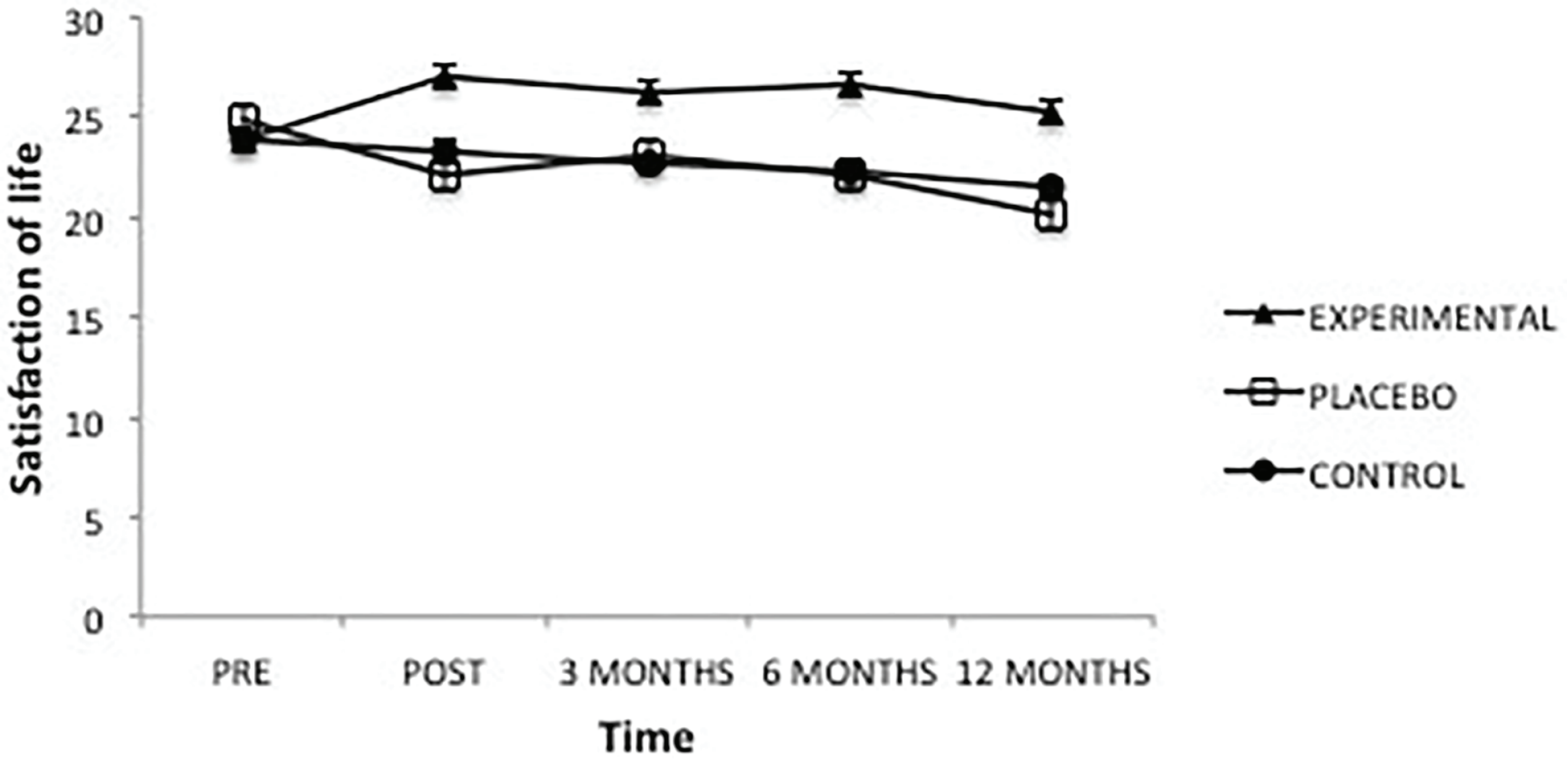
Average scores of experimental, placebo, and control groups in satisfaction with life as a function of time. The error bars represent the standard error of mean (SEM).

In “Satisfaction with life” there was a significant effect from the factor Group *F*(2, 101) = 6.23, *p* = 0.003, $\eta_{p}^{2}=0.11$, from the factor Time *F*(4, 101) = 6.37, *p* = 0.000, $\eta_{p}^{2}=0.06$ and from the interaction between the two *F*(8, 101) = 6.10, *p* = 0.000, $\eta_{p}^{2}=0.11$. Once the intervention was completed, the Experimental group had significantly higher levels of Satisfaction with life than the Placebo (*p* = 0.000) and Control groups (*p* = 0.009); between the latter groups there were no differences (*p* = 0.653). This same result was maintained at 3 months (experimental vs. placebo, *p* = 0.045; experimental vs. control, *p* = 0.024; placebo vs. control, *p* = 1.00), at 6 months (experimental vs. placebo, *p* = 0.002; experimental vs. control, *p* = 0.004; placebo vs. control, *p* = 1.00) and at 12 months after the intervention (experimental vs. placebo, *p* = 0.000; experimental vs. control, *p* = 0.017; placebo vs. control, *p* = 0.738; Figure 3).

Moreover, in the Experimental group scores obtained before the intervention were significantly lower than those obtained afterward (*p* = 0.012) and at 3-month (*p* = 0.013) and 6-month follow-ups (*p *= 0.002), but they disappear after 1 year (*p* = 0.159). The Placebo group presented significantly higher scores before the intervention than after the intervention (*p* = 0.001); also true after 3 months (*p* = 0.021); 6 months; and 12 months (*p* = 0.000); differences between the various post-assessment and follow-on scores were not significant, except for scores after 1 year, which were significantly lower than all other times (post-intervention, *p* = 0.012; 3 months, *p* = 0.000 and 6 months, *p* = 0.000). Finally, in the Control group, pre-intervention scores were significantly higher than those obtained at 6 months (*p* = 0.031) and at 12 months (*p* = 0.001); the latter were lower than those obtained just after the intervention (*p* = 0.042).

**Figure 4:**
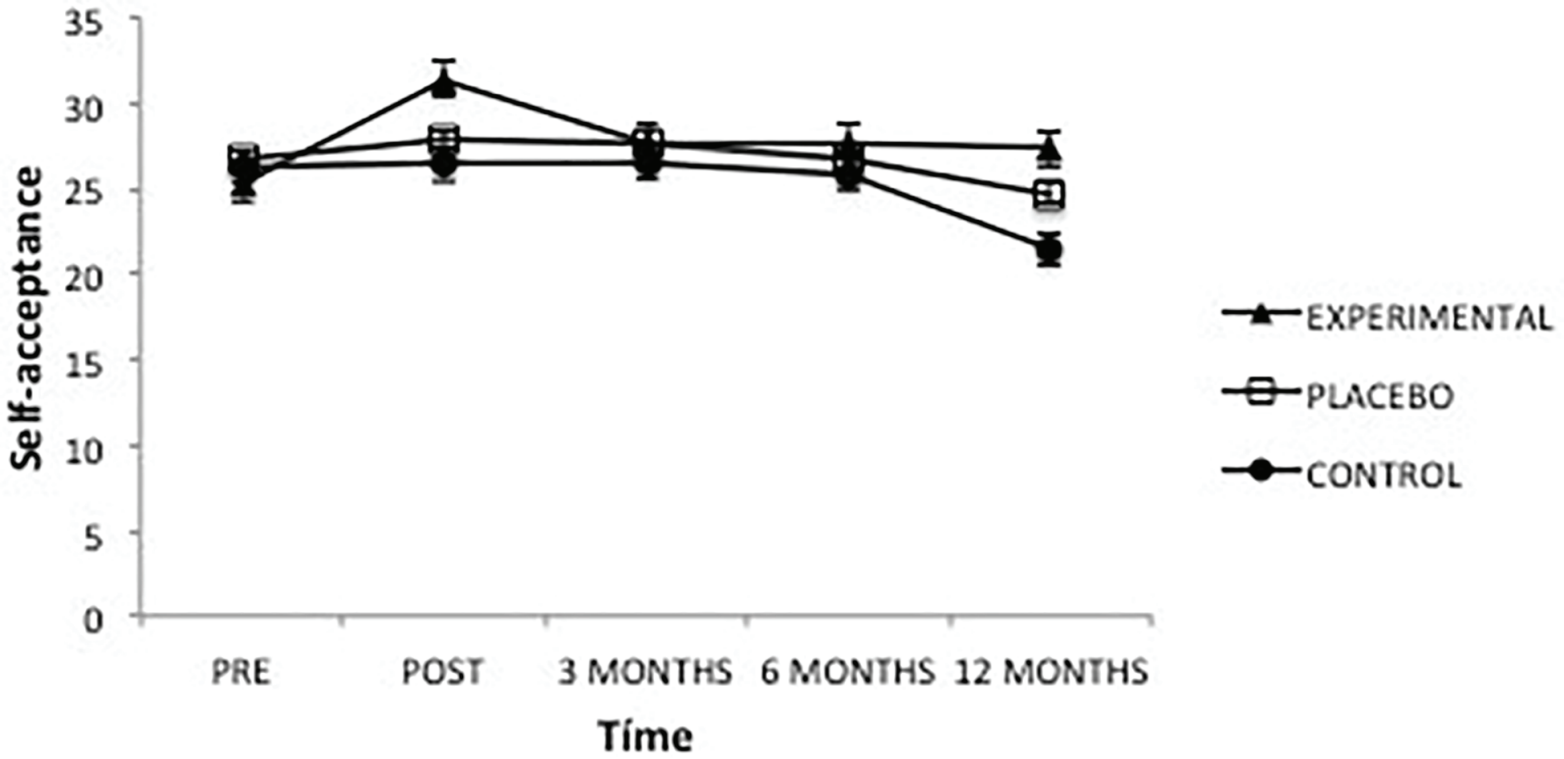
Average scores of experimental, placebo, and control groups in self-acceptance as a function of time. The error bars represent the standard error of mean (SEM).

Regarding the different dimensions of psychological well-being, the analysis of Self-acceptance revealed a significant effect of the factor Time *F*(4, 101) = 8.57, *p *= 0.000, $\eta_{p}^{2}=0.08$ and of the interaction between Time and Group, *F*(8, 101) = 4.02, *p* = 0.000, $\eta_{p}^{2}=0.07$, while there were no significant between-group differences (Figure 4). An analysis of this interaction showed that, once the intervention was completed, the Experimental group had higher levels of Self-acceptance than the Placebo (*p* = 0.010) and Control groups (*p* = 0.000), between which there were no significant differences. These differences disappear, however, 3 months after the intervention.

On the other hand, in the Experimental group, pre-intervention scores were significantly lower than all other measures (post-intervention, *p* = 0.000; 3 months, *p* = 0.014; 6 months, *p* = 0.002 and 12 months, *p* = 0.021), and post-intervention scores were significantly higher than all other times (3 months, *p* = 0.010; 6 months, *p* = 0.009 and 12 months, *p* = 0.009), while there were no differences after 3 months. As for the Placebo group, the only finding was that scores after 12 months were lower than those obtained in post-intervention, *p* = 0.003; 3 months, *p* = 0.001; and 6 months, *p* = 0.032. Finally, in the Control group there were no significant differences between any of the levels of this variable.

**Figure 5:**
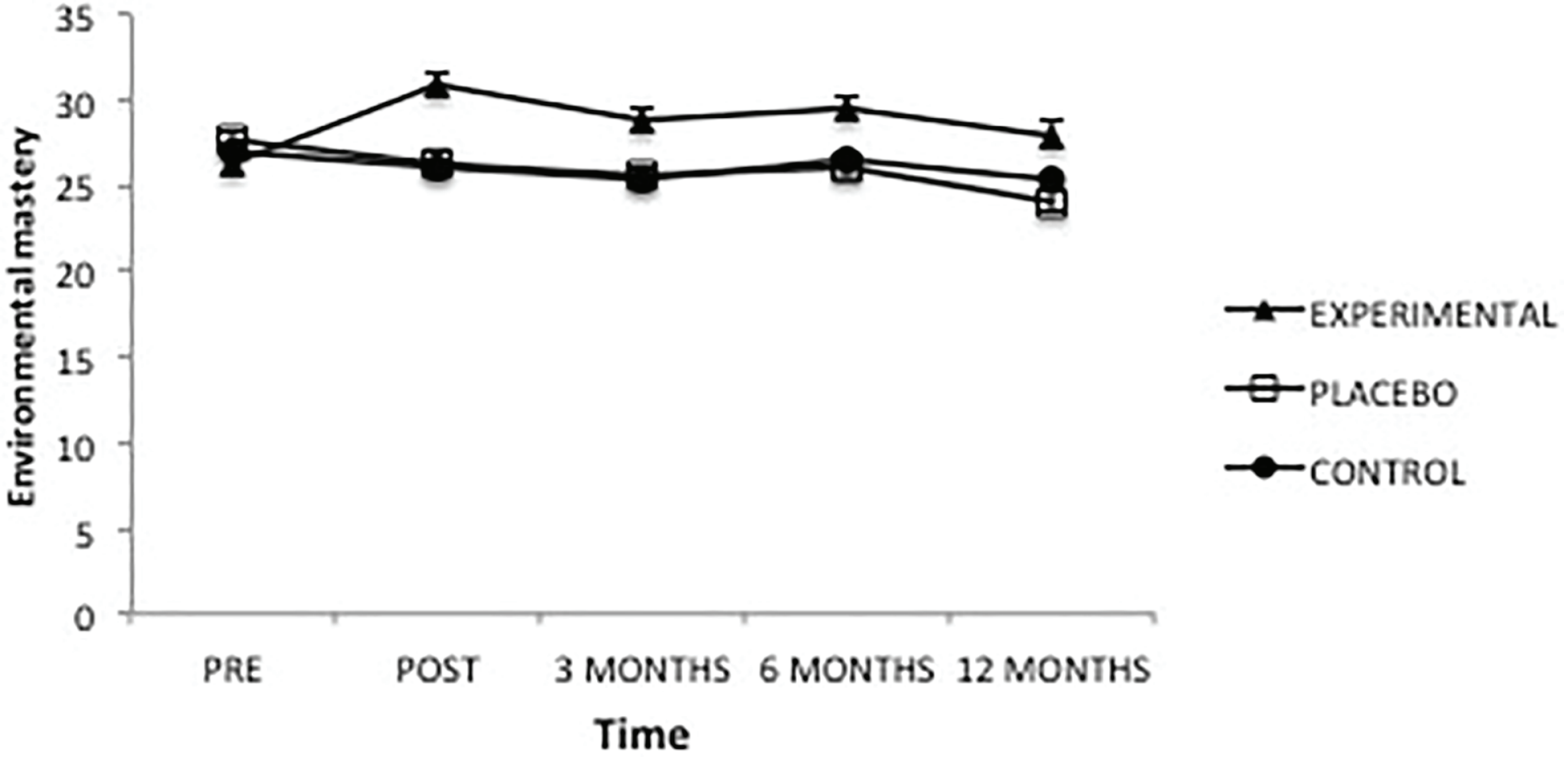
Average scores of experimental, placebo, and control groups in environmental mastery as a function of time. The error bars represent the standard error of mean (SEM).

The analysis of scores obtained in “Environmental mastery” revealed a significant effect from the factor Group *F*(2, 101) = 4.88, *p* = 0.009, $\eta_{p}^{2}=0.09$, from the factor Time *F*(4, 101) = 4.12, *p *= 0.003, $\eta_{p}^{2}=0.04$ and from the interaction *F*(8, 101) = 3.76, *p* = 0.000, $\eta_{p}^{2}=0.07$ (Figure 5). Analysis of this interaction showed that, once the intervention was completed, the Experimental group presented significantly higher levels of Environmental mastery than the Placebo (*p* = 0.001) and Control groups (*p* = 0.000); between the latter groups there were no differences. This same result was maintained at 3 months (placebo, *p* = 0.049; control, *p* = 0.039). At 6 months after the intervention there are only differences between the experimental and placebo groups (*p* = 0.041); after a year the differences had disappeared.

Likewise, in the Experimental group, scores obtained before the intervention were lower than those obtained afterward (*p* = 0.000) or at 3- (*p* = 0.010) and 6-month follow-ups (*p* = 0.000). Similarly, scores following the intervention were found to be greater than those after 3 months (*p* = 0.035) and after 12 months (*p* = 0.003). The same situation occurred between the 6-month and 12-month follow-ups (*p* = 0.021). This shows that environmental mastery increases after the intervention; then, it begins to decline until it returns to its initial values after 1 year. In the Placebo group, there were significant differences between the pre-intervention scores and those obtained at 3 months (*p* = 0.033) and at 12 months (*p* = 0.009), with the pre-intervention scores surpassing the other two levels. Likewise, scores at 12 months were significantly lower than those following the intervention (*p* = 0.021) and then scores at 6 months (*p* = 0.037). Finally, in the Control group no significant differences were found.

**Figure 6:**
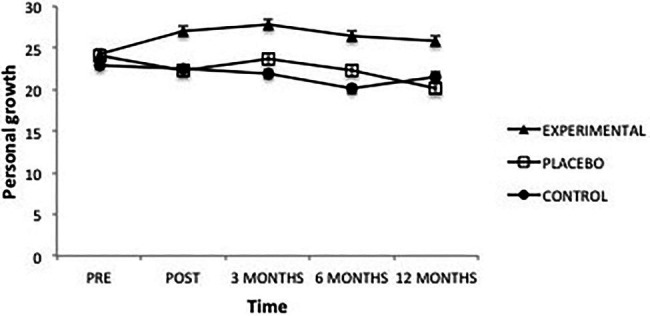
Average scores of experimental, placebo, and control groups in personal growth as a function of time. The error bars represent the standard error of mean (SEM).

Regarding the “Personal growth” dimension, there was a significant effect from Group *F*(2, 101) = 5.71, *p* = 0.004, $\eta_{p}^{2}=0.10$ and from Time *F*(4, 101) = 2.92, *p *= 0.021, $\eta_{p}^{2}=0.03$ as well as from the interaction between the two, *F*(8, 101) = 2.90, *p* = 0.004, $\eta_{p}^{2}=0.05$ (Figure 6). Analysis of this interaction showed that, once the intervention was completed, the Experimental group presented significantly higher levels of Personal growth than the Placebo (*p* = 0.008) and Control (*p* = 0.015) groups; between the latter groups there were no significant differences. This same result was maintained at 3 months (experimental vs. placebo, *p* = 0.047; experimental vs. control, *p* = 0.008), 6 months (experimental vs. placebo, *p* = 0.036; experimental vs. control, *p* = 0.001) and 12 months after the intervention (experimental vs. placebo, *p* = 0.006; experimental vs. control, *p* = 0.049).

On the other hand, in the Experimental group there were significant differences between scores obtained before the intervention, and those obtained afterward (*p* = 0.016) and at 3 months (*p* = 0.016), the initial scores being lower, after this the differences disappear. In the Placebo group, the only difference found was that personal growth at 12 months was significantly lower than before the intervention (*p* = 0.005), and lower than three and 6 months after the intervention (*p* = 0.002). In the Control group, the scores were significantly lower 6 months after the intervention than at all other times (pre-intervention, *p* = 0.002; post-intervention, *p* = 0.009; 3 months, *p* = 0.046 and 12 months, *p* = 0.028), with no other differences found in this group.

**Figure 7:**
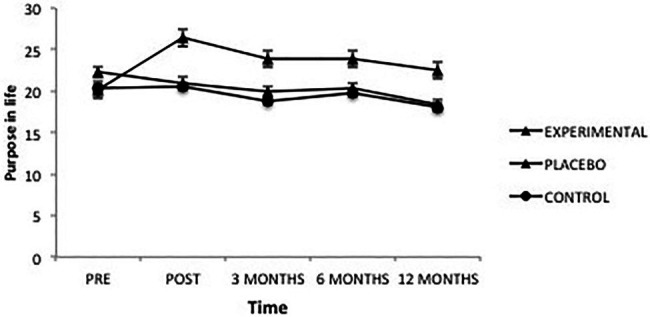
Average scores of experimental, placebo, and control groups in purpose in life as a function of time. The error bars represent the standard error of mean (SEM).

The analysis of scores obtained in “Purpose in life” revealed a significant effect from the factor Group *F*(2, 101) = 4.61, *p* = 0.012, $\eta_{p}^{2}=0.08$, from the factor Time *F*(4, 101) = 7.52, *p *= 0.000, $\eta_{p}^{2}=0.07$ and from the interaction *F*(8, 101) = 5.10, *p* = 0.000, $\eta_{p}^{2}=0.09$ (Figure 7). Once the intervention was completed, the Experimental group had significantly higher levels of Purpose in Life than the Placebo (*p* = 0.001) and Control groups (*p* = 0.000); between the latter groups there were no differences. However, when measured after 3, 6, and 12 months, the differences between the Experimental group and the Placebo group were lost, although the Placebo group scored lower.

Likewise, in the Experimental group, pre-intervention scores were significantly different from the scores obtained after the intervention (*p* = 0.000), and at 3 months (*p* = 0.008), 6 months (*p* = 0.001), and 12 months (*p* = 0.039), with pre-intervention scores lower. On the other hand, post-intervention scores were significantly greater than the other levels (3 months, *p* = 0.026; 6 months, *p* = 0.040; and 12 months, *p* = 0.008). As for the Placebo group, the only differences found were between the 12-month follow-up and the other levels of this variable (pre-intervention, *p* = 0.001; post-intervention, *p* = 0.012; and 6 months, *p* = 0.004), excepting the 3-month follow-up, which did not differ. In the same way, the scores obtained after 3 months were lower than pre-intervention scores (*p* = 0.014). In the case of the Control group, the scores 12 months after the intervention were lower than those at all other times (pre-intervention, *p* = 0.018; post-intervention, *p* = 0.003; and 6 months, *p* = 0.041), except those obtained at 3 months, which did not differ. Similarly, the Control group showed differences between the scores obtained after 3 months and those immediately following the intervention, the latter being higher (*p* = 0.013).

Regarding the dimensions of Positive Relationships with others and Autonomy, the analysis showed no significant effects.

**Figure 8:**
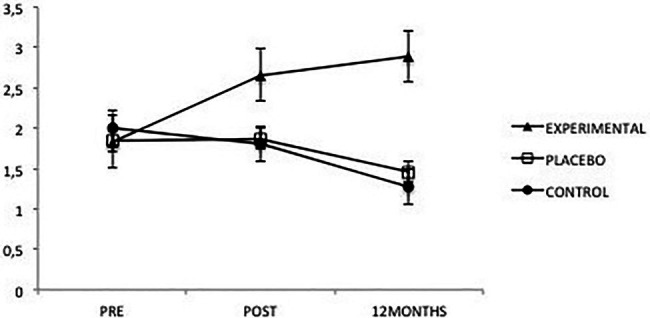
Average scores of experimental, placebo, and control groups in specific positive memories as a function of time. The error bars represent the standard error of mean (SEM).

Regarding memories, positive and negative memories were considered separately, analyzing general and specific memories. The only significant results were found in the case of specific, positive memories, revealing a significant effect from the Group factor *F*(2, 108) = 6.80, *p* = 0.002, $\eta_{p}^{2}=0.11$, and from the interaction between Group and Time *F*(4, 108) = 6.57, *p* = 0.000, $\eta_{p}^{2}=0.11$ (Figure 8). The analysis of the interaction showed that, once the intervention was completed, the Experimental group presented a significantly higher number of specific positive memories than did the Placebo (*p* = 0.033) and Control groups (*p* = 0.021), between which there were no differences. This same result was maintained 12 months after the intervention (*p* = 0.000 for both groups).

On the other hand, the number of memories in the Experimental group was significantly lower before the intervention than afterward (*p* = 0.001) or after 12 months (*p* = 0.000). In the Placebo group, no significant differences were found, and in the Control group, memories before the intervention were significantly greater than 12 months after the intervention (*p* = 0.012).

In addition, the Pearson product–moment correlation coefficient was calculated between specific, positive memories and the rest of the variables, using scores obtained after the intervention. The results showed positive relationships between this type of memories and Subjective Happiness (*r* = 0.44, *p* = 0.000), Satisfaction with Life (*r* = 0.41, *p* = 0.000), Self-acceptance (*r* = 0.27, *p* = 0.004), Positive Relationships with others (*r* = 0.36, *p* = 0.000), Environmental Mastery (*r* = 0.37, *p* = 0.000), Personal Growth (*r* = 0.35, *p* = 0.000), and Purpose in Life (*r* = 0.40, *p* = 0.000). In addition, a negative relationship was found with Depression (*r* = −0.37, *p* = 0.000).

## Discussion and Conclusion

The empirical evidence obtained in this research shows that older adults who receive training in Autobiographical Memory, Gratitude, Forgiveness, and Sense of Humor present a significant increase in Subjective happiness, Satisfaction with life, certain dimensions of Psychological well-being and specific positive memories, as well as a significant reduction in depression. In most cases, the effect on the different measures was maintained for 1 year after the intervention, with exceptions in Subjective happiness and Environmental mastery, where it had disappeared after 6 months, and in Self-acceptance and Purpose of life, where it had disappeared after 3 months. These results show that the adults over age 65 who received this intervention obtained lasting beneficial effects in comparison with persons in the placebo and control groups. On the other hand, in those dependent variables in which the intervention has produced significant results, the effect size values have been medium. We consider this to be an important result since other investigations that have also used these types of activities have generally shown small effect sizes. This result is consistent with the idea that working on autobiographical memory and strengths enhances the effectiveness of this type of intervention in the elderly population.

Similar results were found by Proyer et al. (2014), who carried out an online intervention in Gratitude, Savoring, and the use of Strengths, obtaining an increase in happiness and a decrease in depressive symptoms. In the same way, Ramírez et al. (2014) found reductions in depression and anxiety using training in autobiographical memory, forgiveness, and gratitude. Jiménez et al. (2016) showed that, after a psychoeducational intervention based on sense of humor, gratitude, forgiveness, perseverance, courage, and altruism, the participants significantly increased their level of happiness and reduced their levels of worry, in comparison with a control group whose members did not receive any type of intervention. Ho et al. (2014) obtained a reduction in the number of depressive symptoms and an increase in levels of life satisfaction, gratitude, and happiness, in institutionalized adults, through an intervention focused on optimism, gratitude, happiness, curiosity, altruism, and savoring positive life experiences.

Unlike other studies, the present study took measurements of both subjective and psychological well-being; in other words, this variable was measured from both a hedonic and eudaimonic perspective. One important fact in this regard is that the intervention resulted in a significant increase in several dimensions of psychological well-being: Self-acceptance, Environmental mastery, Personal growth, and Purpose in life. It seems, therefore, that these types of interventions have proven to help older people to feel good about themselves, to choose situations that are favorable for them, to develop their personal capacities and to set new goals in life, bringing about a resulting increase in well-being.

In several studies (Ryff and Keyes, 1995; Keyes et al., 2002), the dimensions of self-acceptance and environmental mastery were found to be related to measures of happiness and satisfaction with life, that is, with measures of subjective well-being, while the dimensions that perhaps most genuinely represent the meaning of psychological well-being – purpose in life and personal growth – show little relation to these types of measures. Our results, however, show relationships existing between all of these, in the same line as in the study by Joshanloo (2019). This discrepancy might be explained by the fact that participants in these studies were from different populations. It is possible, in the case of an elderly population, that the lines between the types of well-being identified in the literature are more blurred.

According to Ryff (1989), one dimension of well-being that remains relatively stable throughout the life cycle is self-acceptance – one of the dimensions most closely related to subjective well-being. Some authors consider this due to it not being affected by physical changes (Benítez Ríos and Domínguez Ortega, 2010). The results of this study, however, do not support these claims. The increase found in this dimension may be due to its very nature. Self-acceptance is more than knowing oneself and having an accurate perception of one’s own actions, motivations and feelings; it also includes attainment of a positive view of oneself.

On the other hand, environmental mastery tends to be greater in older and middle-aged adults than in young people. Nonetheless, the increase that was achieved in our study means that people who have received the intervention can more easily adapt to the inevitable changes in their environment, helping them gain a sense of control. The intervention also brought about a significant increase in the dimensions of purpose in life and personal growth. This fact is important since these dimensions decrease over one’s lifetime, this being particularly pronounced in older adults (Ryff, 1995). In this regard, Navarro et al. (2008) carried out a study exclusively with an elderly population and observed a negative relationship between age and three of Ryff’s dimensions (environmental mastery, personal growth, and purpose in life). These authors indicate that the decrease in environmental mastery possibly has to do with greater use of accommodative strategies, which imply modifying one’s goals, lowering one’s aspirations, abandoning certain objectives, or establishing alternative parameters of comparison with which to evaluate one’s current situation. However, our intervention led to a significant increase in these dimensions, opening up a new line of work, that is, helping older adults set new goals and consider that they are able to carry out projects, by involving them in a continuous process of personal development.

Therefore, our study points in the same direction as did Musich et al. (2018), who found that older people with high levels of well-being have a meaningful and purposeful life thanks to the use of psychological resources that help them face adversities by accepting and giving meaning to their experiences.

Regarding memories, several studies indicate that there is a relationship between depression and overgeneralization of memories. Access to specific autobiographical memories has been shown to be more difficult when people are sad (Philippe et al., 2011). Likewise, older adults have a greater number of general memories and fewer specific memories than young people (Ros et al., 2006), which could be due to a greater presence of affective disorders in the elderly (Navarro et al., 2008). Vázquez and Hervás (2011) postulated that these disorders can alter the interpretation of memories, having negative consequences on satisfaction in older adults, thus affecting their quality of life.

In our study, life review therapy has been used to help people recover and organize their memories, since this therapy is based on recalling specific positive events. Our results show a considerable increase in these memories, and this increase was found to have a significant negative correlation with depression. We therefore observe a relationship between improved mood and the recovery of positive memories. Other studies have found results pointing in the same direction. For example, Preschl et al. (2012) carried out an intervention based on life review therapy and obtained a significant decrease in depression, increase in well-being and decrease in obsessive thoughts. Other studies have found an increase in self-esteem, life satisfaction, psychological well-being, and a decrease in depression (Meléndez Moral et al., 2015; Hitchcock et al., 2017; Jo and An, 2018).

Interventions that lead to recalling specific positive events are thus especially beneficial, since recovery of these memories may be a protective factor against depression (Ramírez et al., 2014; Talarowska et al., 2016), eventually leading to increased life satisfaction (Latorre et al., 2013) and emotional well-being (Bohlmeijer et al., 2007). The present study has found positive relationships between all the dimensions of psychological well-being except for Autonomy, Happiness, Satisfaction with life, and specific positive memories. In this regard, our intervention program is innovative because it can stimulate the recovery of these memories by also including training in gratitude, forgiveness, and a sense of humor, strategies that improve autobiographical recall because they magnify and intensify good memories about the past.

Although our results support the fact that positive interventions help people improve their emotional state and increase their specific memories, the factors that mediate the relationship between depression, gratitude, forgiveness, sense of humor, and memory specificity have not been studied. It would be interesting to discover what those factors are and to what extent each one contributes to the effects obtained. In this regard, several studies suggest that rumination could mediate the relationship between high levels of depression and reduced specific memory (Raes et al., 2012). In fact, rumination is considered to be a risk factor that predicts multiple disorders. Positive activities can disrupt rumination processes by stimulating positive emotions that help people creatively solve their problems. Positive thoughts and behaviors can become more common when a ruminating style is replaced by a more positive style (Layous et al., 2014).

On the other hand, the results obtained in the present investigation represent important empirical support for the fact that training in the strengths of gratitude, forgiveness, and sense of humor is especially effective in this type of population.

Older adults are more likely to value gratitude as a positive, rewarding experience; as we get older we reevaluate our priorities and goals and become more interested in events that we find emotionally pleasing. This may be because older adults realize that they have little time left in their lives, so they focus their resources on what is meaningful and pleasing to them (Layous et al., 2018). This becomes important because experiencing gratitude predicts satisfaction with life, is related to having a purpose in life, and significantly correlates with well-being (Ramírez et al., 2015; García Álvarez et al., 2019). In this regard, Killen and Macaskil (2015) carried out a gratitude intervention with older adults, who wrote daily in a gratitude journal. Stress levels showed a decline, and levels of well-being increased, but these benefits did not persist 1 month after the intervention. The same result was found by Krejtz et al. (2016) and others. Similarly, Salces-Cubero et al. (2019) also found that gratitude training in older persons produced an increase in satisfaction with life, happiness, positive affect, and resilience as well as a decrease in negative affect.

In the area of forgiveness research, it is well known that forgiving is related to both physical and psychological health benefits (Lee and Enright, 2019; Rasmussen et al., 2019). The results obtained here support this statement and are consistent with those found in other research studies. These include Cornish and Wade (2015) and Kent et al. (2018), who found an improvement in psychological well-being; Rey and Extremera (2016), an increase in life satisfaction and reduced depression; Rezaei et al. (2019), an increase in happiness and quality of life; and Sánchez-Cabaco et al. (2019b), a decrease in depression, anxiety and stress.

On the other hand, sense of humor can play a very important role in the well-being of older adults since it contributes to the enjoyment of present events and is a protective factor against adversity and negative emotions (Hone et al., 2015; Tagalidou et al., 2019). Wellenzohn et al. (2016) showed that practicing a sense of humor decreases depression and increases levels of happiness. In the same direction, the studies of Van der Wal and Wok (2019), and Siregar and Gultom (2019) showed that sense of humor reduces depression, anxiety and stress, while Wu et al. (2019), and Zhao et al. (2019) found that a sense of humor leads to increased satisfaction with life and positive affect.

One of the greatest contributions of present study was that effects produced by the program persisted over time. As Sutipan et al. (2017) argue, interventions in positive psychology with people over 60 years of age are beneficial for increasing well-being, happiness, satisfaction with life and for reducing depression, but this benefit is achieved fundamentally in the short term. Research has shown that Life Satisfaction increases with age until the final stage of life is reached; then, a marked decline is observed (Gerstorf et al., 2010). This agrees with the data we obtained from participants in the placebo and control groups: We observed a decrease in the different measures of well-being over time. Using these two groups represents a contribution that endorses the effectiveness of the proposed intervention, given that not all studies use a control group, and very few use a placebo group (Allemand and Flückiger, 2020; Treichler et al., 2020). In our study, it was possible to maintain the benefits obtained from the intervention; lasting benefits are especially important in older adults since this has been associated with higher quality of life (Smith and Bryant, 2016). This achievement may be due to the refresher sessions, which represent a novel aspect in comparison with other programs proposed. We believe that these sessions can maintain the positive emotions produced by the different activities, as the participants have more chances to include them in their daily life. This is a particularly noteworthy aspect of our intervention. On one hand, it is a feature that had not been incorporated in this type of intervention to date, and on the other hand, as our results shown, it enabled the intervention benefits to be maintained as long as 1 year after the intervention. This represents an important contribution, because most studies have not carried out follow-up for this length of time, and when they did, effects were not maintained (e.g., Westerhof and Slatman, 2019).

Authors, such as Lyubomirsky and Layous (2013) have suggested that the increase in positive emotions may be one mechanism that mediates the effectiveness of positive interventions. As indicated in studies by Fredrickson et al. (2005) and Fredrickson (2013), positive emotions can directly affect health and well-being. Positive emotions produce an accumulation of growth and expansive effects, which can even transform individuals, making them healthier, more socially integrated, more effective, and resilient. They predict an increase in well-being by expanding the individual’s psychological resources. This undoubtedly may have helped the participants in this study to more positively cope with the events that have occurred in their lives; the positive emotions produced by the intervention activities can become a component of the coping process, where situations are considered as challenges rather than threats (Folkman, 2008).

Despite the contributions we have made here, our investigation presents a number of limitations. First, the sample selection was not random, so there could be issues in generalizing the results, although the sample does represent the demographic characteristics of the elderly in Spain. Second, only subjective measurements were taken. Future studies should include objective measures, such as behavioral measures, evaluation of facial expressions and even objective measures of health. Third, the measurement of memories was taken at two times only: immediately following the intervention and after 12 months, due to its complexity and to the characteristics of the study participants. Nonetheless, the intervention clearly proved to be effective in this measure. Fourth, while the results have shown the intervention to be effective, we cannot know whether some activities produced more benefits than others. This will have to be investigated in the future in order to better match the intervention to the characteristics of this population. Finally, any changes that the intervention produced in forgiveness, gratitude, and sense of humor were not measured.

Future studies should incorporate refresher sessions to corroborate their usefulness in maintaining intervention benefits over time. In addition, both the interventions and the refresher sessions could be conducted using information technologies. In fact, so-called positive technologies make use of informational technology to improve the quality of one’s personal experience; their goal is to increase well-being and generate strengths and resilience in individuals, organizations, and society (Riva et al., 2012; Drozd et al., 2014; Barceló Soler et al., 2019).

Health professionals have more and more resources to help people construct their well-being. This means improving quality of life, preventing the appearance of psychopathologies, developing emotional competencies – in short, making people happier, regardless of their age.

## Data Availability Statement

The raw data supporting the conclusions of this article will be made available by the authors, without undue reservation.

## Ethics Statement

The studies involving human participants were reviewed and approved by Comité de Bioética de Andalucía. Junta de Andalucía. Spain. The patients/participants provided their written informed consent to participate in this study.

## Author Contributions

AC-G, ER-F, and AO-M contributed to conception and design of the study. AC-G organized the database and wrote the first draft of the manuscript. AO-M and ER-F performed the statistical analysis and wrote sections of the manuscript. All authors contributed to the article and approved the submitted version.

## Conflict of Interest

The authors declare that the research was conducted in the absence of any commercial or financial relationships that could be construed as a potential conflict of interest.

## Publisher’s Note

All claims expressed in this article are solely those of the authors and do not necessarily represent those of their affiliated organizations, or those of the publisher, the editors and the reviewers. Any product that may be evaluated in this article, or claim that may be made by its manufacturer, is not guaranteed or endorsed by the publisher.
